# Necrology

**Published:** 1901-09

**Authors:** 


### NECROLOGY.

Dr. A. M. Rook, inspector of the Bureau of Animal Industry, died at La Junta, Col., May 19th last. Dr. Rook was engaged in the sheep-inspection division, and had been associated with the bureau for some time.

Dr. H. M. Burgess, graduate of the Veterinary Department, Harvard University, class of 1897, died at Covington, La., June 6, 1901. Dr. Burgess was attached to the inspection department of the Bureau of Animal Industry, and had held several positions at a number of points in connection with the work of that department.

Dr. Aaron E. Shaub, of Lancaster, Pa., died suddenly at his home, August 8, 1901. He was stricken fatally when returning from his stable. Dr. Shaub was a practitioner in Lancaster County for upward of thirty years, and was the father of Drs. Elmer and J. K. Shaub, both graduates in veterinary medicine of the American and United States Colleges respectively. The deceased conducted a large veterinary hospital in connection with his practice, and was favorably known throughout Lancaster and adjoining counties.

